# Transport phenomena in complex systems (part 2)

**DOI:** 10.1098/rsta.2021.0366

**Published:** 2022-02-21

**Authors:** Dmitri V. Alexandrov, Andrey Yu. Zubarev

**Affiliations:** Department of Theoretical and Mathematical Physics, Laboratory of Multi-Scale Mathematical Modeling, Ural Federal University, Ekaterinburg 620000, Russia

**Keywords:** transport phenomena, complex systems, heterogeneous materials, metastable and non-equilibrium systems, phase transformations, biophysical systems

## Abstract

This theme issue, in two parts, continues research studies of transport phenomena in complex media published in the first part (Alexandrov & Zubarev 2021 *Phil. Trans. R. Soc. A* **379**, 20200301. (doi:10.1098/rsta.2020.0301)). The issue is concerned with theoretical, numerical and experimental investigations of nonlinear transport phenomena in heterogeneous and metastable materials of different nature, including biological systems. The papers are devoted to the new effects arising in such systems (e.g. pattern and microstructure formation in materials, impacts of external processes on their properties and evolution and so on). State-of-the-art methods of numerical simulations, stochastic analysis, nonlinear physics and experimental studies are presented in the collection of issue papers.

This article is part of the theme issue ‘Transport phenomena in complex systems (part 2)’.

## 1. Introduction

Equilibrium and non-equilibrium transport phenomena accompanied with phase transformations, pattern formation, rheological effects and other non-equilibrium processes in natural and artificial complex systems and materials (biological tissues and living cells; composite materials; multicomponent polymers and melts, etc.) are attracting considerable interest from researchers, engineers and bio-engineers because they are widespread in nature and actively used in progressive high industrial and biomedical technologies [1–9]. The features of transport phenomena in many real complex and disordered systems (biological tissues, composite materials, etc.) can be found in their multicomponent, multistability, strong interactions between the components, and various synergetic effects. Very often the high reaction of these systems to external electrical and magnetic fields cannot be explained by the classical thermodynamics of single-component systems [10]. The opportunity to control the internal architecture and macroscopical properties of these systems with the help of external physical fields and temperatures opens up promising ways to create materials with fundamentally new functions and to organize the necessary effects (hyperthermia, tissue growth and regeneration) in biological media and materials. Despite the differences in their physical natures, many of these phenomena can be studied by using analogical theoretical, computational and experimental methods, although the specifics of each of these processes require the development of original approaches. Complicated by various physical, chemical and biological processes, the entire physical spectrum of transport phenomena is presented in two parts of this theme issue: magnetohyperthermia processes, the magneto-active reaction of polymers, colloids, endosomal network dynamics in living cells, particle growth in metastable liquids, microstructural formation during dendritic evolution and the like. The effect of the kinetics of internal structuring on macroscopic phenomena and the functional characteristics of systems is the focus of this issue. The similarities in the approaches used when studying physically different phenomena are demonstrated and discussed. The presented works are at the cutting edge of research into synergetic phase and structural formations in complex multifunctional disordered systems. These works build upon the laws of the physics of complex systems: they extend this field, highlighting the challenge of non-equilibrium phenomena in multicomponent inorganic and biological matter. The importance of this theme is defined by the application of the presented results to biological and medical technologies. Modern biophysics, as well as new biomedical applications for cancer and brain stroke therapy, requires the development of theoretical foundations for understanding and describing these phenomena in biological and organic media (e.g. drug address delivery; the phase transformations in proteins and insulins; modelling and performing diagnostics of the bloodstream in branched vessels; magnetohypethermia therapy of cancers [11–16]). The issue is focused on the development of theories and their computational and experimental verification in order to create a new foundation for future biomedical applications. This explains why the issue is timely.

The present issue covers the rapidly developing research area of multicomponent materials with complex disordered internal structures (non-equilibrium systems such as metastable and biological ones, magneto-active soft and liquid systems and noise-sensitive systems). It covers multiple disciplines, ranging from the non-equilibrium statistical physics of microscopic effects to phenomenological non-equilibrium thermodynamics, condensed matter physics and biomedical applications. To present a complex picture of micro- (nano) and meso-temporal and spatial scales in transport phenomena, a study of various heterogeneous materials (from metastable and multicomponent liquids to biological cell cultures) is included in this issue. Special attention is paid to developing the scientific background of biomedical applications (the intensification of address drug delivery in thrombosed blood vessels; intercellular transport for controlling the import and distribution of cargo into a series of endosomes; magnetic hyperthermia as a method of cancer therapy; haemodynamics in branched coronary arteria; and non-contact diagnoses of stenosis). Experimental and theoretical studies of non-equilibrium and field-provoked structural and phase transformations are included. Also, we have included articles devoted to noise-induced phenomena in non-equilibrium systems, which essentially change their deterministic behaviour [17,18].

## 2. The general content of the issue

**Figure 1.**
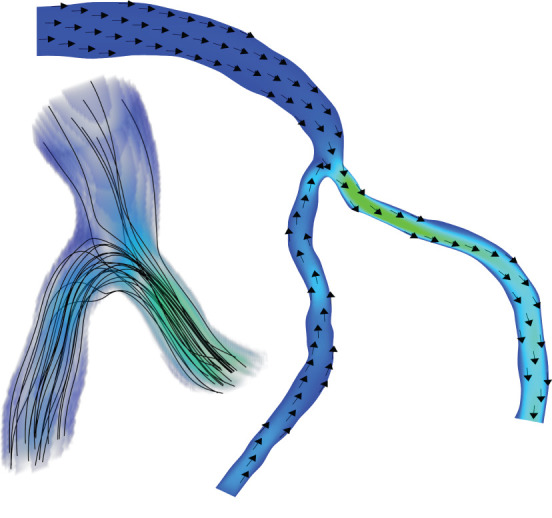
Blood velocity and streamlines in the bifurcation area before the vessel stenting (see, for details, [19]). (Online version in colour.)

**Figure 2.**
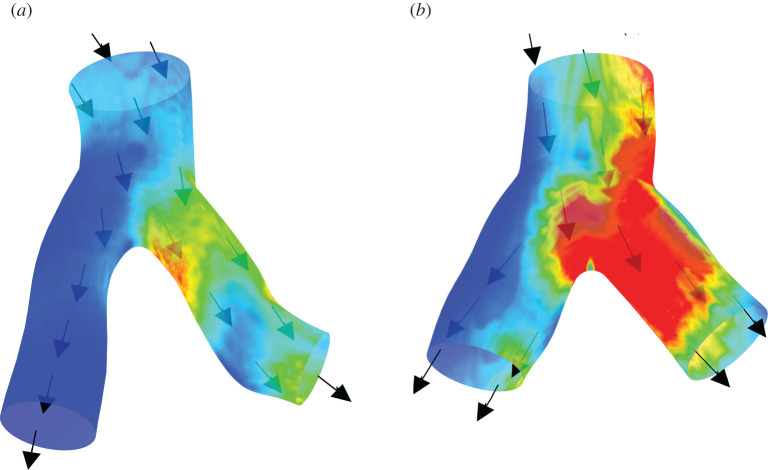
Tangential stresses at different times of the cardiac cycle near the bifurcation site of the left coronary artery before (*a*) and after (*b*) stenting (see, for details, [19]). (Online version in colour.)

Transport phenomena in multicomponent complex and disordered systems are widespread in nature and actively met in many industrial and biomedical technologies. The structural transformations and non-equilibrium phenomena in these systems (for instance, the solidification of metastable melts and alloys; rheological patterns in multicomponent fluids; noise-provoked patterning; structural transformations in field-sensitive materials) affect and determine their macroscopic properties and behaviour (see, for example, figures 1 and 2, where hydrodynamic structuring in blood vessels with stenosis is shown). These transformations occur during different phase transformations, ranging from materials physics to biophysics and the life sciences (for example, hydrodynamic flows in porous materials and mushy layers met in material science and geophysics) [20–29]. By using various external impacts (magnetic and electrical fields; temperature gradient; noise; hydrodynamic flows), one can tune the evolution of these transformations, the final results of phase transitions, and the observed macroscopic behaviour of the studied systems. Recent studies have shown that in many important situations, phase transitions and non-equilibrium transport in complex systems cannot be described within the framework of traditional and classical theories. Biological, thermal, mechanical and other non-equilibrium systems can be very sensitive to external stochastic impacts; noise-induced phase transitions very often take place in these systems [30–32]. Magnetic fields provoke the clusterization of ferromagnetic nanoparticles, dramatically change the physical and mechanical properties of magnetic suspensions and polymers, and induce circulating flows and hydrodynamic patterns that can intensify drug delivery in blood vessels and other biological tissues. These phenomena have applications in cancer and insult therapy and regenerative medicine: as such, the development of an accurate method of quantitative description is required. Studies on these transport phenomena have opened up new and challenging fields in the physics of condensed matter and smart materials, as well as in biophysics. In the present issue, the problems of equilibrium, non-equilibrium, and noise-induced phase transitions and transport and relaxation phenomena in metastable and biologically active systems, field-sensitive materials, soft composites and biological tissues are considered.

So, this issue is organized as follows. Transport phenomena during phase transitions leading to various patterns and microstructure formation are studied in works [33–36]. These studies are continued in the papers [37–40], where multiscale modelling of such phenomena based on the phase-field, phase-field crystal, molecular dynamics and cellular automata methods has been carried out. The effects of external processes such as magnetic fields and hydrodynamic flows on transport peculiarities have been analysed in [19,41–43]. In addition, applications of nonlinear haemodynamics to myocardial revascularization are studied in [19] with special attention to the influence of bifurcation lesions in coronary arteries. The effects of stochastic forcing and, in particular, coloured-noises, on transport phenomena and formation of complex oscillatory regimes, are shown in studies [44,45].

## 3. Conclusion

The theoretical and experimental papers, as well as numerical simulations of nonlinear transport phenomena collected in this special issue, will have a great impact on future research in this scientific field (e.g. peculiarities of transport processes when a heterogeneous system transforms into a disordered state, transport in bioactive and chemically reactive media, stochastically induced transport during transitions from order to chaos and back). Studying the nonlinear transport of heat, dissolved impurities and particles in such complex media will make it possible to investigate the internal structure of new generations of materials and establish new laws of matter transport in living systems (for example, intracellular transport of nanoparticles and their distribution inside living cells, endosomal network dynamics) or systems exposed to external influences (electromagnetic field, hydrodynamic flows, external noise). This, in turn, will make it possible to develop a theoretical basis for transport processes in complex non-equilibrium, metastable, disordered or self-organizing systems in non-living and living matter, such as mysterious glass transitions, transitions from order to chaos and back, transport of medicinal compounds in human and animal blood vessels, anomalous and nonlinear intracellular transport, and so on.

## Data accessibility

This article has no additional data.
